# α-Dystrobrevin knockout mice have increased motivation for appetitive reward and altered brain cannabinoid receptor 1 expression

**DOI:** 10.1186/s40478-022-01434-4

**Published:** 2022-08-31

**Authors:** Cheryl A. Hawkes, Christopher J. Heath, Matthew M. Sharp, Dariusz C. Górecki, Roxana O. Carare

**Affiliations:** 1 Division of Biomedical and Life Sciences, Lancaster University, Lancaster, UK; 2 School of Life, Health and Chemical Sciences, Open University, Milton Keynes, UK; 3 Clinical and Experimental Sciences, University of Southampton, Southampton, UK; 4 Biomedical Imaging Unit, University Hospital Southampton NHS Trust, Southampton, UK; 5 Molecular Medicine, School of Pharmacy and Biomedical Sciences, University of Portsmouth, Portsmouth, UK

**Keywords:** α-Dystrobrevin, Appetitive motivation, Endocannabinoid, Brain

## Abstract

α-Dystrobrevin (α-DB) is a major component of the dystrophin-associated protein complex (DAPC). Knockout (KO) of α-DB in the brain is associated with astrocytic abnormalities and loss of neuronal GABA receptor clustering. Mutations in DAPC proteins are associated with altered dopamine signaling and cognitive and psychiatric disorders, including schizophrenia. This study tested the hypothesis that motivation and associated underlying biological pathways are altered in the absence of α-DB expression. Male wildtype and α-DB KO mice were tested for measures of motivation, executive function and extinction in the rodent touchscreen apparatus. Subsequently, brain tissues were evaluated for mRNA and/or protein levels of dysbindin-1, dopamine transporter and receptor 1 and 2, mu opioid receptor 1 (mOR1) and cannabinoid receptor 1 (CB1). α-DB KO mice had significantly increased motivation for the appetitive reward, while measures of executive function and extinction were unaffected. No differences were observed between wildtype and KO animals on mRNA levels of dysbindin-1 or any of the dopamine markers. mRNA levels of mOR1were significantly decreased in the caudate-putamen and nucleus accumbens of α-DB KO compared to WT animals, but protein levels were unaltered. However, CB1 protein levels were significantly increased in the prefrontal cortex and decreased in the nucleus accumbens of α-DB KO mice. Triple-labelling immunohistochemistry confirmed that changes in CB1 were not specific to astrocytes. These results highlight a novel role for α-DB in the regulation of appetitive motivation that may have implications for other behaviours that involve the dopaminergic and endocannabinoid systems.

**Supplementary Information:**

The online version contains supplementary material available at 10.1186/s40478-022-01434-4.

## Introduction

α-dystrobrevin (α-DB) is a principal component of the dystrophin-associated protein complex (DAPC), which also comprises β-DB, dystrophin, dystroglycans, sarcoglycans, and other intracellular proteins [1]. The DAPC is important for cellular anchoring to the extracellular matrix [2]. Although initially described in skeletal muscle, α-DB is also expressed in multiple organs, particularly at blood-tissue barriers in the testes, stomach, lungs, inner ear and brain [3, 4]. In the brain, α-DB is present in neurons, astrocytes, pial vessels and endothelial cells [5, 6]. The DAPC-like complex in neurons helps to stabilize synapses and anchor GABA_A_ receptors at specialised sites in the post-synaptic membrane [7]. In astrocytes, α-DB interacts with syntrophin, dystrophin protein 71 and the DAPC to secure aquaporin-4 channels within the plasma membrane and attach astrocyte endfeet to the basement membrane [7].

Dysfunction of DAPC proteins is associated with various forms of muscular dystrophy, including Duchenne muscular dystrophy (DMD) [8]. In addition, mutations in α-DB are associated with familial Ménière's disease, which is characterised by tinnitus and sensorineural hearing loss [9, 10]. Loss of α-DB in astrocytes is associated with increased blood–brain barrier permeability and progressive edema, as well as thickening of the basement membrane and reduced intramural periarterial clearance of the β-amyloid protein [11, 12]. Double knockout of α-DB and β-DB in cerebellar Purkinje neurons results in decreased number and size of GABA receptor clusters and behavioural motor deficits [6].

In addition to auditory and muscular dysfunction, cognitive impairment and behavioural disorders have been identified in up to 50% of people with muscular dystrophies [7]. The *mdx* mouse model of DMD shows progressive impairments in learning and memory, elevated anxiety-related behaviours, and deficits in the passive avoidance task [13, 14]. Recently, dysbindin-1 (also termed dystrobrevin-binding protein 1), which binds to α- and β-DB in the DAPC [15], has been identified as a susceptibility gene for schizophrenia [16] and polymorphisms in the dysbindin-1 gene are associated with altered emotional working memory [17]. However, the behavioural consequences of α-DB knockout in the brain have not yet been evaluated.

Risk alleles of dysbindin-1 and reductions in the dysbindin-1 protein are associated with altered dopamine (DA) signaling and accumulation of cell surface D2 receptors [18, 19]. DA is a key regulator of motivation for rewarding stimuli, including hedonistic foods [20]. This is regulated via dopaminergic projections that originate in the ventral tegmental area (VTA) and synapse in the nucleus accumbens (nAc) and limbic lobe (mesolimbic pathway), basal ganglia (mesostriatal pathway) and prefrontal cortex (PfCtx) (mesocortical pathway) [21]. Appetitive motivation is broadly underpinned by distinct but related states of ‘wanting’ and ‘liking’ of a food reward. ‘Wanting’ is associated with the incentive salience attributed to rewarding stimuli and related cues and is associated with subjective food cravings in humans [22]. ‘Liking’ refers to the hedonistic feeling that is generated by the consumption of palatable foods. Thus, the pleasurable feeling ascribed to cues associated with rewarding stimuli can generate behaviours to seek out and consume the stimuli [22]. Activation of the mesolimbic pathway is associated with motivation for hedonistic foods, while the mesocortical pathway regulates the emotional response to feeding [20]. In addition to DA, motivation for appetitive reward is influenced by the opioid and endocannabinoid (eCB) systems. In particular, administration of mu-opioid receptor agonists stimulates the intake of high-fat foods and ingestion of palatable food stimulates release of opioids [23]. Similarly, eCB concentrations are increased following consumption of rewarding foods and modulation of cannabinoid receptor 1 (CB1) activity moderates the release of DA, opioids and other neurotransmitters that regulate reward-related behaviour [24].

In light of the previously reported associations between alterations in DAPC proteins, cerebrovascular dysfunction and DA signaling, the purpose of this study was to characterise the behavioural consequences of α-DB knockout on motivation and reward and to evaluate associated molecular and cellular changes in the DA, eCB and opioid systems.

## Materials and methods

### Animals

Male homozygous α-DB knockout mice (B6;129-Dtna ^tm1Jrs^/J, α-DB KO) and C57Bl/6 wildtype (WT) control littermates were generated from a heterozygous α-DB KO breeding colony purchased from Jackson labs (Strain #:010976) and maintained at the University of Southampton. Animals were transferred to the Open University and left to acclimate for 2 weeks. Mice were group housed throughout the experiment with the exception of 4 days during which food and water intake were assessed in individually housed mice. Animals were kept on a standard 12-h light/dark cycle (lights on at 7am) and allowed food and water ad libitum until the start of the behavioural testing. All procedures were carried out in accordance with the regulations of the University of Southampton and the Open University AWERB and the Home Office (P12102B2A and PPL 70/8507).

### Apparatus and reagents

Behavioural testing began when animals were 12–14 weeks old (n = 12/group) using Bussey-Saksida Mouse Touch Screen Chambers (Campden Instruments, Loughborough, UK). The same animals were used in all behavioural tests and the experimenters remained blinded to animal genotype throughout testing. Mice underwent food restriction to ~ 90% of free-feeding weight beginning one week before the start of the tests and were kept on restriction for the duration of testing. Strawberry milkshake (Yazoo®, FrieslandCampina UK, Horsham, UK) was used as the appetitive operant reinforcer (20 μL/reward). All animals first underwent 2 sessions of habituation (20 min/session with milkshake present) to the chambers before the start of behavioural testing.

### Fixed and Progressive ratio task (FR/PR)

Touchscreen FR and PR schedules were carried out in 12–14 weeks old animals as described previously [25]. In brief, mice underwent 1 day of initial touch (15 trials over 60 min), 1 day of FR1 (15 trials over 60 min), 1 day of FR2 (15 trials over 60 min) and 2 days of FR5 (6 trials over 60 min) training. After completion of FR5, animals were initially evaluated in a PR4 schedule (2 days, 30 trials over 60 min). However, as the majority of both WT and α-DB KO were still responding at the end of the PR4 session, animals were then progressed through an abbreviated PR8 (1 day, 30 trials over 60 min) and a PR12 (3 days, 30 trials over 60 min) testing session before finally running the mice on a PR16 schedule (3 days, 30 trials over 60 min). In each case, the reward response in the PR sessions was increased on a linear + n basis upon completion of each trial (e.g. PR16 = 1, 17, 33 touches per reward). If no response was detected within 5 min, the session was terminated, and the number of target responses emitted in the last completed trial was used as the breakpoint. Mice were tested over 3 sequential days and the breakpoints were averaged.

### Pairwise visual discrimination (PVD) task

The PVD task was carried out as previously reported [26] when mice were 17–19 weeks old. During the discrimination acquisition period, mice were presented with two stimuli, one of which was designated as the rewarded (conditioned) stimulus. If mice selected the incorrect stimulus, they were held in a correction trial in which the spatial presentation of the correct and incorrect stimuli was kept constant until the mouse made a correct response. Otherwise, left/right stimuli presentation was pseudorandomized within each trial. Mice completed 30 trials over 60 min until they achieved ≥ 80% correct response over 2 consecutive days. All mice reached this criterion within 8 days, after which they underwent 2 refresher sessions (30 trials over 60 min) during which they again had to achieve ≥ 80% correct response in each session to progress to the reversal training. One WT and one α-DB KO mouse were omitted from reversal training after failing to reach criteria in the refresher sessions. After discrimination acquisition, animals underwent reversal training during which the correct and incorrect stimuli were reversed. The mice were tested for another 10 days or until they again achieved ≥ 80% correct response. Days to criteria in both the acquisition and reversal sessions were averaged and reversal sessions were also categorized according to the number of trials in which performance was < 50% correct or ≥ 50% correct [27].

### Effort-related choice (ERC) task

The ERC task was carried out when the animals were 20–22-weeks-old using similar parameters to the FR task, with some modifications. At the start of each ERC session, three pre-weighed food pellets were placed randomly into the chamber. Mice were given the choice to eat the pellets and/or complete the trials to collect the milkshake reward and the weight of the food pellets was recorded at the end of the session. The reward response requirement was increased progressively from ERC16, ERC32 and ERC64. Mice underwent 5 consecutive days of testing in each schedule and the number of completed trials were averaged. After completion of ERC64, mice were rested for 1 day and then tested on the FR64 task for 2 days without any food in the testing chamber.

### Extinction task

At 24–26-weeks old, mice were first trained to complete 30 FR1 trials for 3 consecutive days. Subsequently, the animals underwent an extinction phase, in which the same screen stimulus disappeared when touched or after 10 s of presentation, and no reinforcement tone or milkshake was provided. The extinction task was carried out over 12 days and the number of completed trials in each session was recorded.

### Intake of food, water and milkshake

After completion of the extinction task, food-restricted mice (26–28 weeks old) were placed into a fresh cage containing pre-weighed food pellets and randomly allocated to also receive water or milkshake. This was repeated the next day, with mice given the opposite liquid such that all mice were exposed to the food + water and food + milkshake combination. Mice were allowed to consume the food and liquid for 60 min, after which the intake was recorded. Subsequently, food restriction was terminated, and mice were allowed *ad lib* food and water consumption for 5 days. Data collected for food and water consumption was recorded by cage and averaged across the number of animals per cage. Subsequently, mice were singly housed and given *ad lib* access to milkshake in their home cages for 24 h and intake per mouse was recorded. Mice were returned to group housing with their original cagemates and kept on *ad lib* diet until sacrifice 6 weeks later.

### Tissue collection

Following completion of the behavioural testing, the same mice were killed at 32–34-weeks of age with an overdose of sodium pentobarbitone (20% w/v; Animalcare, York, UK). All animals underwent cardiac perfusion with 0.01 M phosphate buffered saline (PBS) and tissues from mice in each genotype were randomly assigned to be collected as fresh frozen or perfusion fixation. For RT-qPCR and Western blots, brain tissues were then rapidly removed and dissected for prefrontal cortex (PfCtx), caudate-putamen (Cpu), nucleus accumbens (nAc), tegmentum and cerebellum. Tissues were snap frozen on dry ice and kept at −80 ºC until use. For immunohistochemistry, mice were additionally perfused with 4% paraformaldehyde (PFA), and brains were removed and kept in 4% PFA overnight at 4 °C. The next day, brains were washed in 0.01 M PBS and stored in 30% sucrose until being sliced on a cryostat (20 μm thickness), collected in a free-floating manner and stored at −20 °C until use.

### RT-qPCR

Frozen tissues (n = 7/group) were immersed in RNAlater-ICE (Fisher Scientific, Loughborough, UK), RNA was extracted using the RNeasy kit (Qiagen, Manchester, UK) and cDNA was synthesised using the Applied Biosystems™ High-Capacity cDNA Reverse Transcription Kit (Fisher Scientific). mRNA levels of dystrobrevin binding protein 1 (*Dtnbp1*), dopamine transporter (*Scl6a3*), dopamine receptor 1 (*Drd1a*), dopamine receptor 2 (*Drd2*), cannabinoid receptor 1 (*Cnr1*), mu opioid receptor 1 (*Oprm1*) and β-actin (*Actb*) (KiCqStart® SYBR® Green Primers, Merck, Gillingham, UK) were measured using the QuantiTect SYBR Green PCR Kit (Qiagen). Genes of interest were normalised to β-actin and the relative expression between WT and α-DB KO mice was quantified using the 2^–∆∆Ct^ method.

### Western blotting

Frozen brain tissues (n = 7/group) were processed by gel electrophoresis as previously described [28]. Membranes were incubated with primary antibodies against cannabinoid receptor 1 (CB1, 1:1000, Abcam, Cambridge, UK), mu opioid receptor 1 (mOR1, 1:1000, Merck) and dopamine transporter (DAT, 1:750, Merck). Blots were stripped and re-probed with anti-glyceraldehyde-3-phosphate dehydrogenase (GAPDH, 1:50,000, Merck) antibody to ensure equal protein loading. Two blots were used to generate data for each antibody. Immunoblots were quantified by densitometry using Fiji (NIH, Maryland, USA) and calculated as an optical density ratio of protein levels normalized to GAPDH levels and expressed as % of WT values.

### Immunohistochemistry

Brains were processed for immunohistochemistry (n = 5/group) as described previously [28]. Brain sections were washed in 0.01 M PBS, blocked with a mixture of 7.5% normal goat serum + 7.5% normal donkey serum and then incubated overnight at 4 ºC with anti-GFAP (1:1500; Abcam), anti-CB1 (1:1000, Abcam) and anti-NeuN (1:750, Abcam). The next day, sections were incubated with anti-rabbit AlexaFluor555, anti-chicken AlexaFluor 633 and anti-mouse AlexaFluor488 (1:200 each, 2 h at room temp, Fisher Scientific UK). Images were captured on an LM880 Zeiss confocal microscope (Cambridge, UK). For 3D reconstruction, images were deconvolved using AutoQuant X3 (MediaCybernetics Inc, Rockville MD) and then processed using Imaris software (Bitplane, Oxford, UK).

### Statistical analysis

Data was checked for normality using the Kolmogorov–Smirnov test. The ROUT test was used to identify and exclude outliers. Analyses between WT and KO mice were carried out using two-tailed Student’s t-test or a Mann–Whitney U test where data were not normally distributed. Comparison of multiple factors were carried out using two-way ANOVA (with repeated measures when the same animals were tested multiple times), with Sidak post-hoc test using GraphPad Prism (San Diego, USA). Rate of learning during acquisition and reversal in the PVD task was analysed by log-rank (Mantel-Cox) test. Data represent mean ± SEM and *p* < 0.05 was considered to be statistically significant.

## Results

### α-DB KO mice show increased responding in the PR schedule

**Fig. 1:**
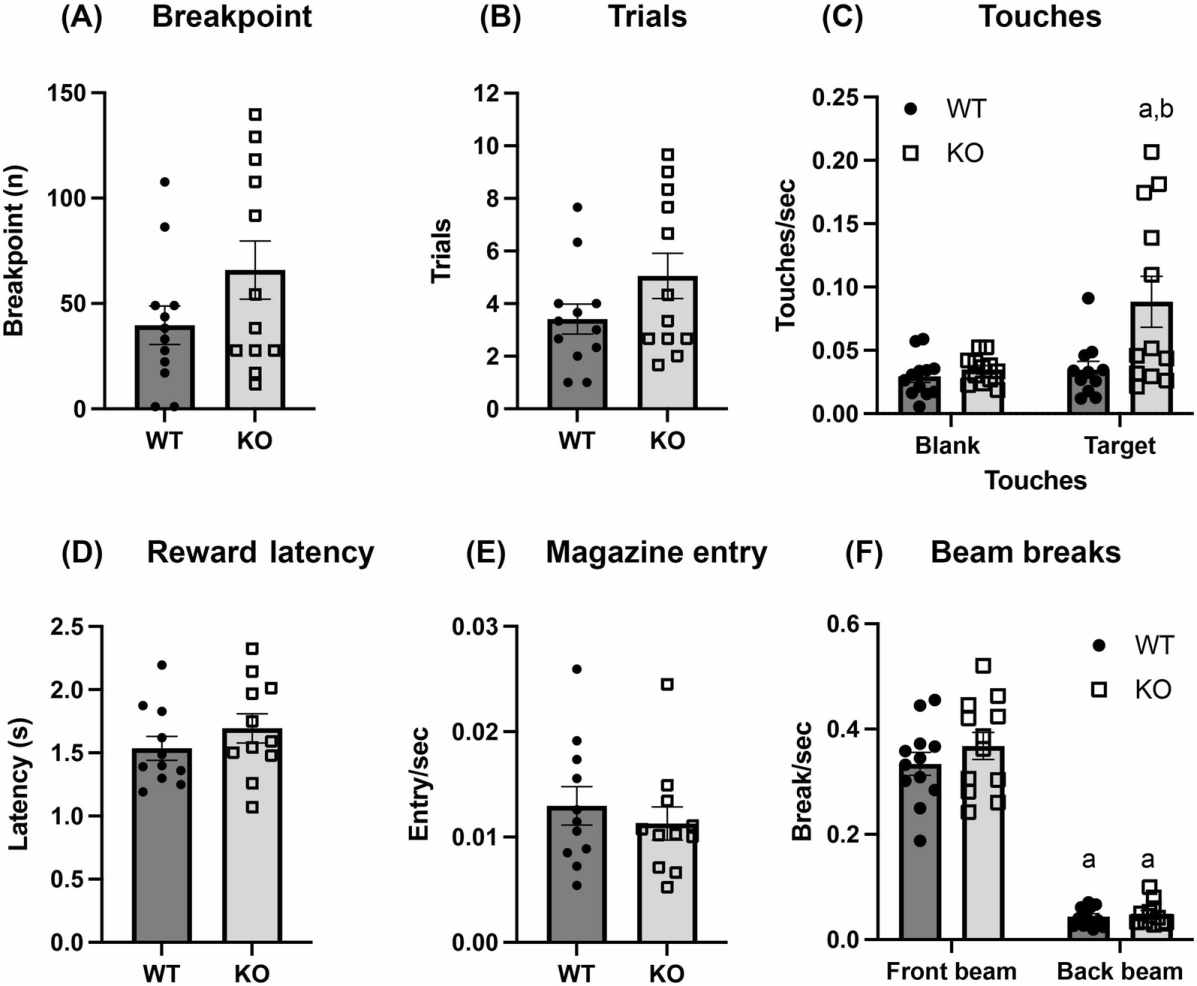
Behavioural outputs of WT and α-DB KO mice on the PR16 task. α-DB KO mice showed a non-significant trend towards increased breakpoint (**a**) and completed trials (**b**) compared to WT animals. Target touches and target vs. blank touches were significantly higher in KO than WT mice (**c**). No differences were observed between α-DB KO and WT animals on reward collection latency (**d**), number of magazine entries/sec (**e**) or front and rear beam breaks/sec (**f**). n = 12/group. For C, a = *p* < 0.01 vs. blank touches/sec, b = *p* < 0.01 vs. WT. For F, a = *p* < 0.0001 vs. front beam breaks/sec, two-way repeated measures ANOVA with Sidak’s post-hoc

To first determine the motivation of WT and α-DB KO mice for an appetitive reward, mice were tested on a PR task. Initial testing on a PR4 schedule found that KO mice showed significantly higher breakpoints than WT animals (U = 33, *p* = 0.04; Additional file 1: Figure S1A). However, we observed that 67% of WT and 100% of KO animals were still responding at the end of the PR4 session. Therefore, animals were transitioned systematically through PR8, PR12 and PR16 schedules to find a work requirement where the majority of mice stopped responding by the end of the session (Additional file 1: Figure S1B and C). α-DB KO mice showed significantly higher breakpoint than WT mice in PR12 (t(21) = 2.2, *p* = 0.04; Additional file 1: Figure S1B), however responding remained above 50% for KO mice (Additional file 1: Figure S1C). Persistent responding decreased to 33% and 42% of WT and KO mice, respectively, in PR16 which was deemed to be sufficiently demanding. α-DB KO mice had a higher breakpoint and greater number of completed trials in PR16 compared to WT animals, although this was not statistically significant due to higher spread of responses in this schedule (breakpoint: t(22) = 1.6, *p* = 0.13; trials: t(22) = 1.6, *p* = 0.13; Fig. 1a, b). Target touches/sec were significantly higher in KO vs. WT mice (genotype: F(1,43) = 7.1, *p* = 0.01, post hoc *p* = 0.003) and target vs. blank touches/sec were also higher in KO mice (touches: F(1,43) = 6.9, *p* = 0.01, post hoc *p* = 0.003), while WT animals made the same number of target and blank touches in PR16 (touches: F(1,43) = 6.9, *p* = 0.01, post hoc *p* = 0.93; Fig. 1c). A significant genotype x touch effect was also observed (F(1,43) = 4.7, *p* = 0.04). No differences were observed between animal groups in average latency to collect reward (t(20) = 1.1, *p* = 0.3; Fig. 1d) or magazine entries/sec (U = 48, *p* = 0.44; Fig. 1e). Both WT and KO mice made significantly more front beam breaks/sec compared to rear bream breaks/sec (breaks: F(1,22) = 386.1, *p* < 0.0001, all post hocs *p* < 0.0001), however no differences in beam breaks were noted between WT and KO animals (genotype: F(1,44) = 1.3, *p* = 0.27; Fig. 1f).

### α-DB KO mice do not show alterations in executive function, but do exhibit some perseverative behaviour

**Fig. 2:**
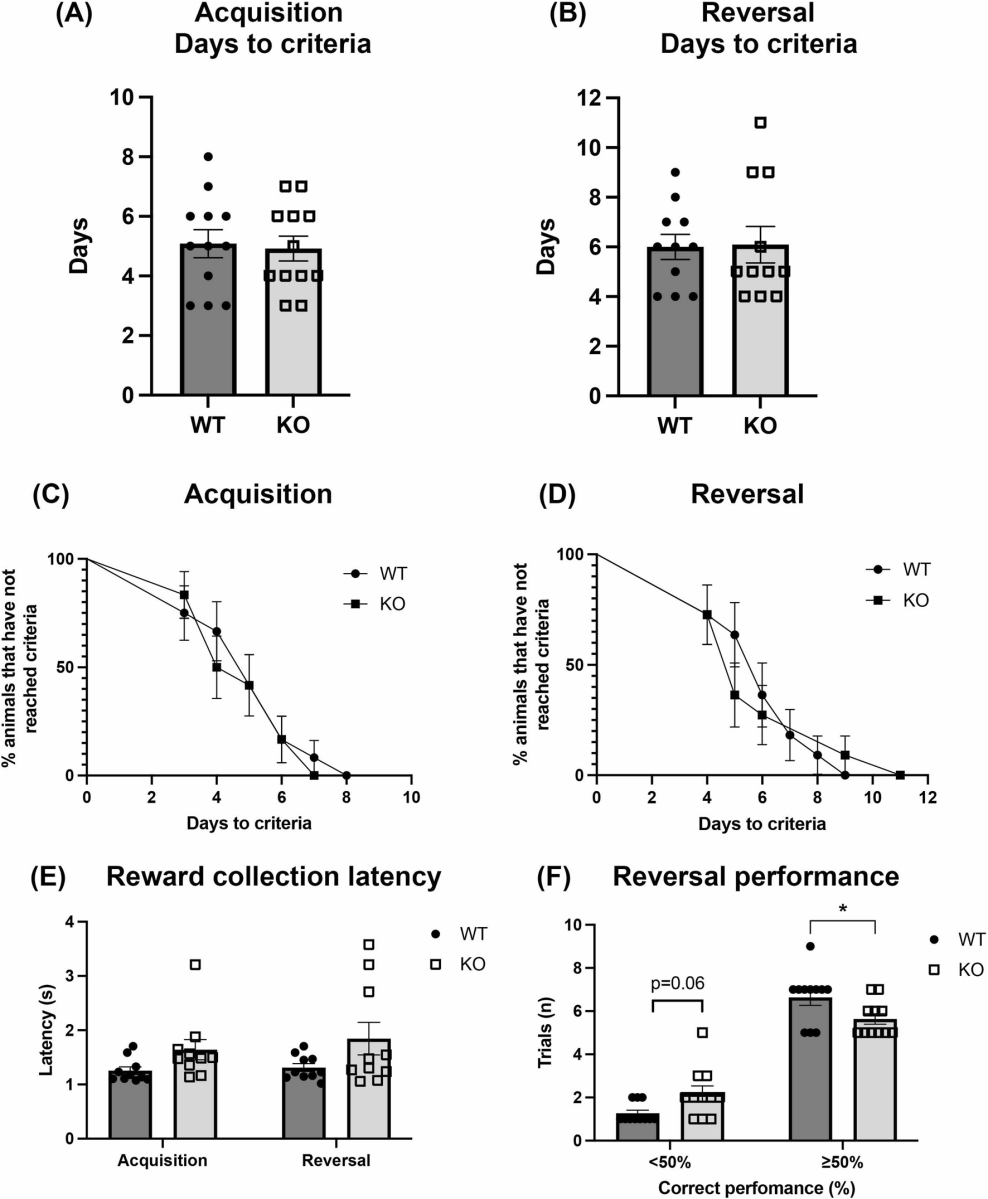
Behavioural outputs of WT and α-DB KO mice on the PVD task. No significant differences were observed between α-DB KO mice and WT mice on days to criteria (correct identification of rewarding stimulus in ≥ 80% of trials) during acquisition (**a**) and reversal (**b**) of the task. In addition, the rate of learning, as determined by the daily % of animals that have not reached criteria, was similar between WT and KO mice in both acquisition (**c**) and reversal (**d**). Reward collection latency did not differ between WT and KO mice in either phase of training (**e**). The number of trials in which performance was < 50% correct and ≥ 50% correct during the reversal sessions differed between WT and KO mice (**f**). n = 12/group. For **a** and **b**, *p* > 0.05, two-tailed t-test. For **c** and **d**, *p* > 0.05, log-rank (Mantel-Cox) test. For **e** and **f**, *p < 0.05, two-way repeated measures ANOVA with Sidak’s post-hoc

To determine if the increased response rate of the KO animals in the PR schedule was due to perseveration [29], mice were then tested on the PVD task. As shown in Fig. 2, all animals successfully reached criteria on both learning and reversal. No difference was seen between WT and α-DB KO mice on the number of days to criteria in either the acquisition (t(22) = 0.27, *p* = 0.79) or reversal phase (U = 56, *p* = 0.79). The rate of learning and reward collection latency during acquisition and reversal was also similar between the animal groups (learning: χ2 (1) = 0.117, *p* = 0.74; latency: F(1,18) = 0.15, *p* = 0.75); Fig. 2c–e). Analysis of the number of below- and above-chance errors during the reversal phase showed a significant genotype x error interaction (F(1, 20) = 13.7, *p* = 0.001) and post hoc analysis showed that KO mice completed significantly fewer above-chance trials (p = 0.04) and showed a non-significant trend (*p* = 0.06) towards more below-chance trials than WT mice (Fig. 2f).

### Increased response of α-DB KO in ERC schedule

**Fig. 3:**
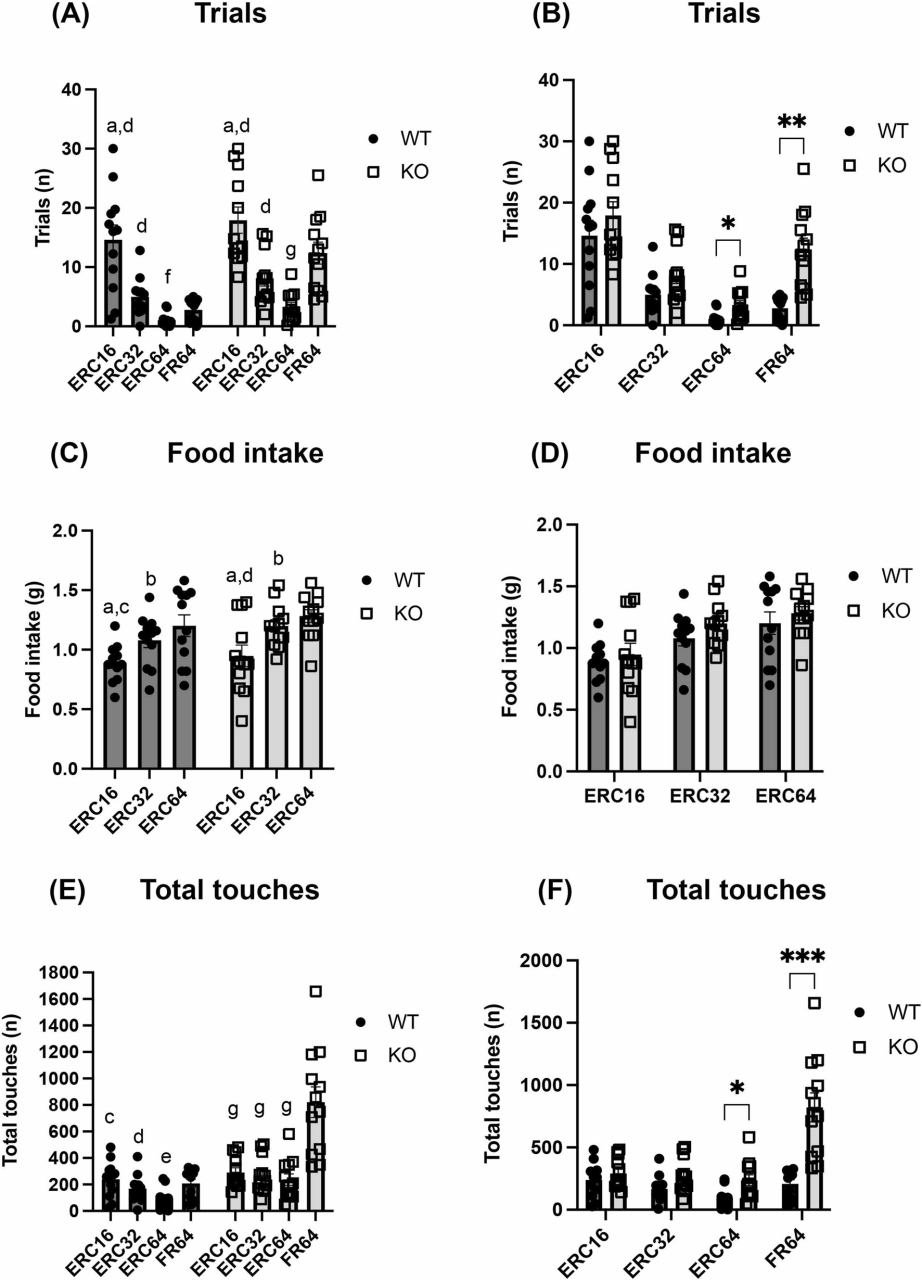
Behavioural outputs of WT and α-DB KO mice on the ERC task. Both WT and α-DB KO mice completed significantly fewer trials between ERC16, ERC32 and ERC64 (**a**). The number of completed trials increased significantly between ERC64 and FR64 in both mouse groups (**a**). In addition, α-DB KO mice completed significantly more trials than WT mice on the ERC64 and FR64 tasks (**b**). The amount of food consumed during the trial was significantly increased between the ERC16, ERC32 and ERC64 schedules in both WT and KO mice (**c**). Food intake did not differ between WT and α-DB KO mice during any of the ERC tasks (**d**). Total touches decreased significantly between ERC16, ERC32 and ERC64 in WT mice but remained stable in KO animals (**e**), while KO mice made more touches than WT animals in ERC64 and FR64 (**f**). n = 12/group. For within-group comparisons (**a**, **c**, **e**), a = *p* < 0.01 vs. ERC32, b = *p* < 0.05 vs. ERC64, c = *p* < 0.01 vs. ERC64, d = *p* < 0.001 vs ERC64, e = *p* < 0.05 vs. FR64, f = *p* < 0.01 vs. FR64, g = *p* < 0.001 vs FR64, two-way repeated measures ANOVA with Sidak’s post-hoc. For between-group comparisons (**b**, **d**, **f**), *p < 0.05, **p < 0.01, ***p < 0.001 vs KO, two-way repeated measures ANOVA with Sidak’s post-hoc

To more fully explore the increased motivation of α-DB KO mice, the same animals were then tested in the ERC task. As expected, the number of completed trials decreased significantly in both the WT and KO groups as the work requirement to receive a reward increased across the ERC schedules (schedule: F(1.78, 39.1) = 60.1, *p* < 0.0001; genotype F(1, 22) = 7.5, *p* = 0.01, schedule x genotype: F(3, 66) = 4.9, *p* = 0.004; all post hocs *p* < 0.05; Fig. 3a, b). This corresponded with a significant increase in food consumption in the testing chamber in both groups (schedule: F(1.42, 31,2) = 36.5, *p* < 0.0001, all post hocs *p* < 0.05), and WT and KO animals ate equal amounts of food (genotype: F(1, 22) = 1.02, *p* = 0.32; Fig. 3c, d). In the ERC64 schedule, α-DB KO mice completed significantly more trials than WT animals (genotype: F(1, 22) = 7.5, *p* = 0.01, post hoc *p* = 0.03; schedule x genotype F(3. 66) = 4.9, *p* = 0.004; Fig. 3b). Moreover, whereas WT mice showed a progressive decrease in total touches between ERC16, ERC32 and ERC64 (schedule: F(1.33, 29.3) = 33.7, *p* < 0.0001, all post hocs *p* < 0.01 vs. ERC64), KO mice maintained a constant number of touches between all ERC schedules (*p* > 0.05 for all post hocs) and made significantly more touches than WT mice in ERC64 (genotype: F(1,22) = 15.2, *p* = 0.0008, post hoc *p* = 0.03; schedule x genotype: F(3,66) = 23.8, *p* < 0.0001; Fig. 3e, f).

To confirm that the decreased engagement with the ERC task was due to effortful choice, mice were then tested on an FR64 schedule without food pellets in the chamber. Both WT and KO mice completed significantly more trials (schedule: F(1.78, 39.1) = 60.1, *p* < 0.0001, all post hocs *p* < 0.01; Fig. 3a, b) and made more total touches in the FR64 task compared to ERC64 (schedule: F(1.33, 29.3) = 33.7, *p* < 0.0001, all post hoc *p* < 0.05; Fig. 3e, f). In addition, KO mice made more total touches (genotype: F(1, 22) = 15.2, *p* = 0.0008, post hoc *p* = 0.0009) and completed more trials than WT mice during FR64 (genotype: F(1,22) = 7.5, *p* = 0.01, post hoc *p* = 0.001), suggesting a higher motivation for the milkshake (Fig. 3b, f). No difference in latency to collect reward (genotype: F(1,16) = 3.77, *p* = 0.07), front beam breaks/sec (genotype: F(1,18) = 0.37, *p* = 0.55), rear beam breaks/sec (genotype: F(1,22) = 0.38, *p* = 0.54) or magazine entries/sec (genotype: F(1,21) = 0.77, *p* = 0.39) was observed between WT and KO in any of the ERC schedules (Additional file 2: Figure S2A-C).

Because α-DB KO mice maintained a consistent number of touches over the ERC schedules despite decreasing frequency of milkshake reward, mice were then progressed through an extinction protocol to determine if they would continue to engage with the task in the absence of appetitive stimuli. A significant decrease in the number of completed trials was observed by day 4 of extinction in both animal groups (time: F(5.02, 110.4) = 58.3, *p* < 0.0001, all post hocs *p* < 0.001 from day 4–12 vs. day 1), but no differences were noted between WT and KO mice on any day of the protocol (genotype: F(1,22) = 1.95, *p* = 0.18; Additional file 2: Figure S2D).

### Fully fed α-DB KO mice show increased ad lib intake of milkshake

**Fig. 4:**
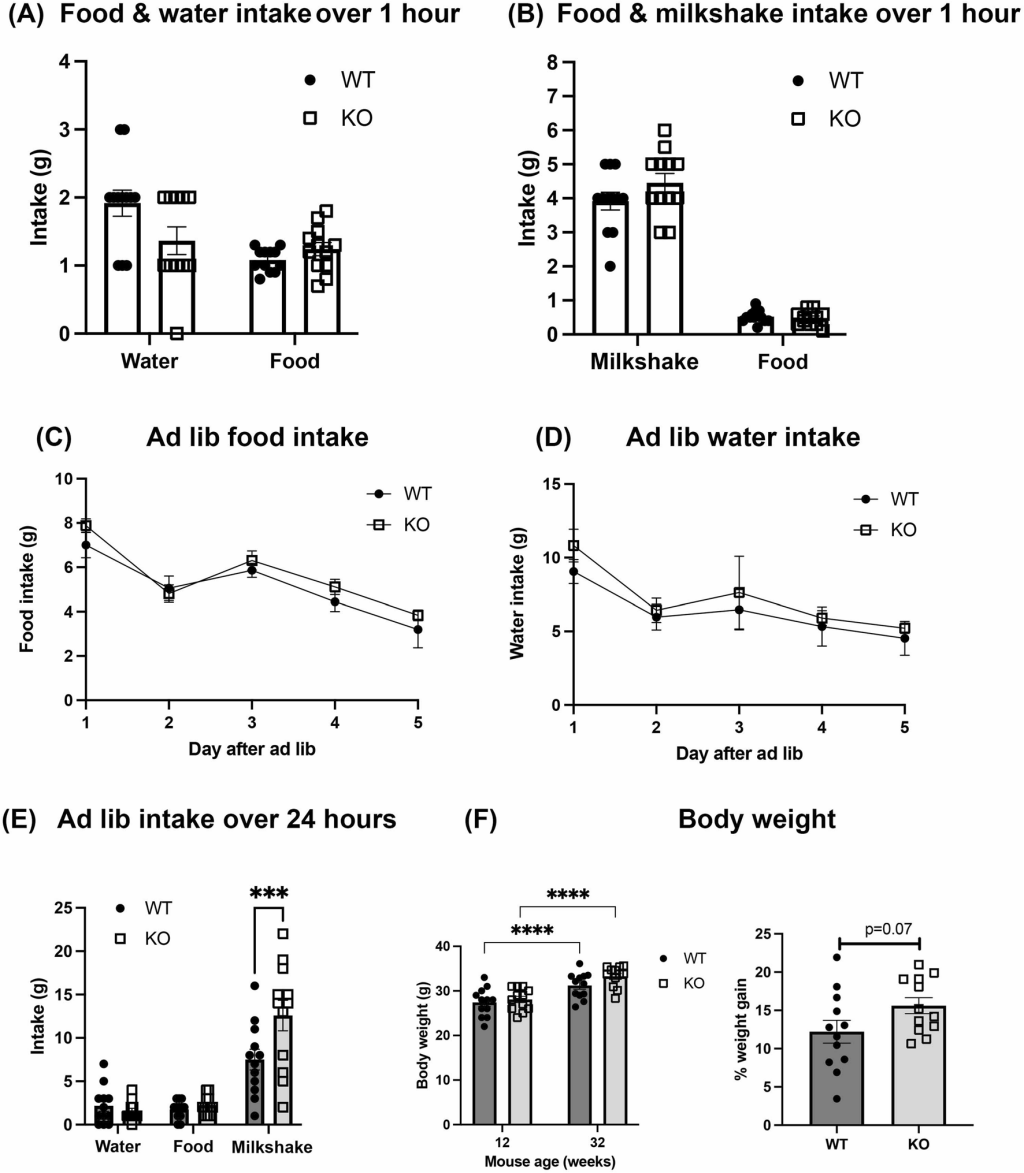
Intake of food, water and milkshake during and after food restriction. Food-restricted mice were allowed to consume food pellets and water (**a**) or food pellets and milkshake (**b**) for 1 h. WT and α-DB KO mice consumed the same amount of food, water and milkshake during this period. Intake of food and water did not differ between WT and KO animals allowed ad libitum access (**c** and **d**). Fully fed α-DB KO mice drank significantly more milkshake than WT mice over a 24-h period (**e**). Body weight at the start (12 weeks old) and end (32 weeks old) of the experiment did not differ between WT and KO mice, although both groups gained a significant amount of weight over time (**f**). KO mice showed a trend towards greater weight gain between the start and end of the experiment compared to WT animals (**f**). n = 12/group. For **a**–**e**, *p* > 0.05, two-way repeated measures ANOVA. For **f**, ****p < 0.0001, two-way repeated measures ANOVA with Sidak’s post-hoc

To determine if the increased motivation for the milkshake reward was due to a generally elevated appetite, food-restricted mice were allowed to freely consume food pellets, water or milkshake over a 60 min period. Both WT and KO mice drank significantly more milkshake than water (food type: F(3,87) = 177.6, *p* < 0.001, all post hocs *p* < 0.0001; genotype x food type: F(3,87) = 3.5, *p* = 0.02; Fig. 4a, b), but there were no differences between animal groups on food, water or milkshake intake (genotype: F(1,87) = 0.05, *p* = 0.83). To evaluate if food restriction was masking potential genotype differences, animals were then put back on *ad lib* feeding for 5 days, following which they were allowed access to milkshake for 24 h alongside food and water in their home cage. Daily food and water intake per cage was similar between WT and KO mice (food: F(1,5) = 0.65, *p* = 0.46; water: F(1,5) = 0.25, *p* = 0.64; Fig. 4c, d). However, α-DB KO animals drank significantly more milkshake than WT mice (genotype: F(1,65) = 4.5, *p* = 0.04, post hoc *p* = 0.0009; genotype x food type: F(2, 65) = 5.1, *p* = 0.009; Fig. 4e). Comparison of mouse body weight at the start and end of the experiment did not differ between animal groups (genotype: F(1,22) = 13.6, *p* = 0.22), although both WT and KO mice gained a significant amount of weight over the course of the experiment (time: F(1,22) = 236.9, *p* < 0.0001, all post hoc *p* < 0.0001; time x genotype: F(1,22) = 5.7, *p* = 0.03) and there was a non-significant trend (*p* = 0.07) for greater weight gain over the course of the experiment in the KO mice (Fig. 4f).

### Levels of CB1 expression are altered in α-DB KO mice

**Fig. 5:**
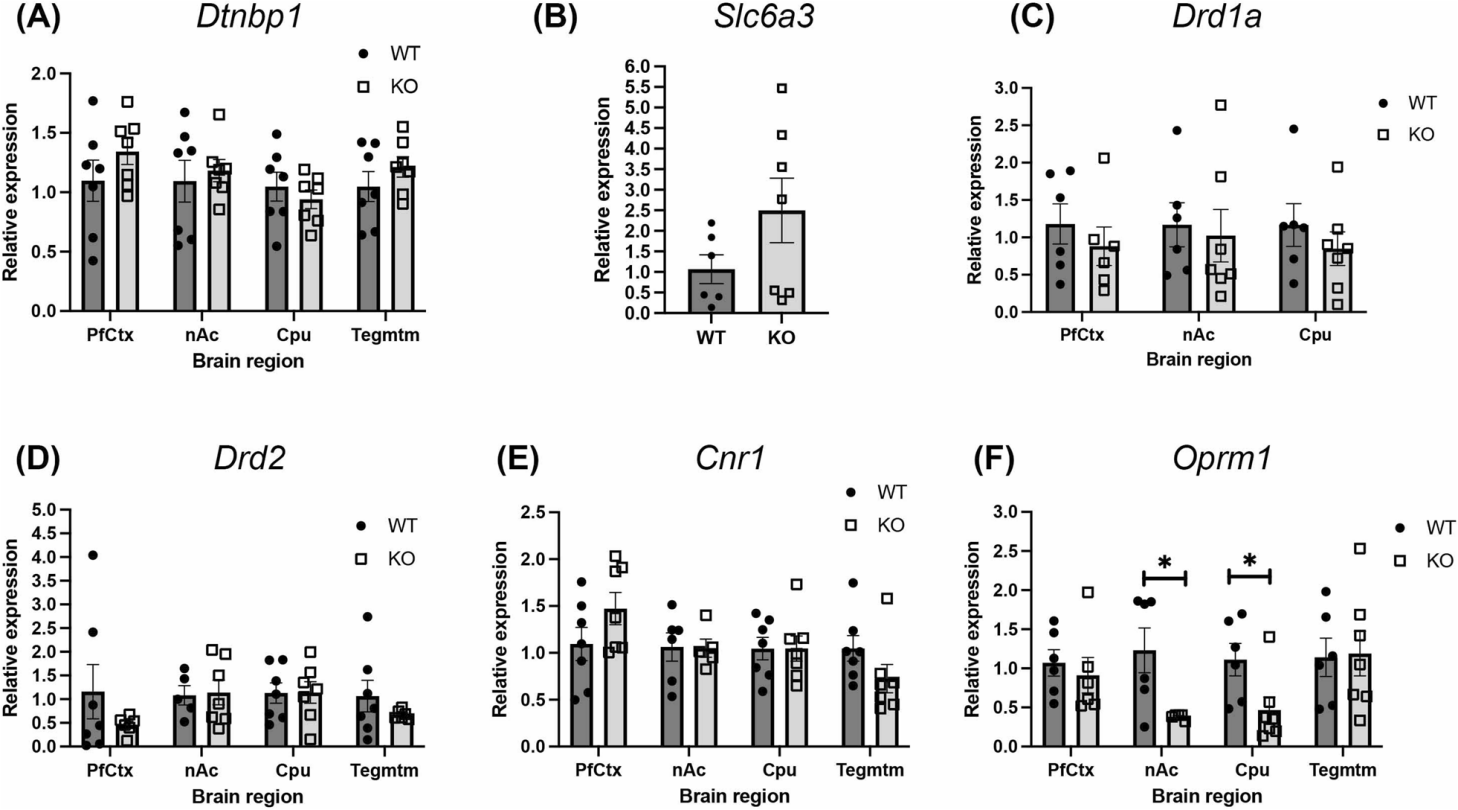
mRNA levels of dystrobrevin binding protein 1, dopamine, mu opioid and cannabinoid receptors. No differences were noted between WT and α-DB KO mice in mRNA levels of dystrobrevin binding protein 1 (*Dtnbp1*) (**a**), dopamine transporter (*Scl6a3*) (**b**), dopamine receptor 1 (*Drd1a*) (**c**), dopamine receptor 2 (*Drd2*) (**d**) or cannabinoid receptor 1 (*Cnr1*) (**e**). mu Opioid receptor 1 (*Oprm1*) levels were significantly decreased in the nucleus accumbens (nAc) and caudate-putamen (Cpu) of KO mice relative to WT animals (**f**). PfCtx = prefrontal cortex, Tegmtm = tegmentum. *p < 0.001, two-way ANOVA with Sidak’s post-hoc

To determine if the increased motivation in the α-DB KO mice was due to alterations in dysbindin-1 or in the DA, opioid and cannabinoid systems, mRNA levels of *Dtnbp1, Scl6a3, Drd1a, Drd2, Oprm1* and *Cnr1* were evaluated by RT-qPCR in the PfCtx, Cpu, nAc and/or tegmentum of WT and KO mice. As shown in Fig. 5a, no differences were observed in *Dtnbp1* expression between mouse groups in any brain region (genotype: F(1,48) = 1.2, *p* = 0.27). *Scl6a3* expression, which was only reliably detected in the tegmentum, did not differ significantly between mouse groups, although 4 of the 7 KO mice showed a > twofold increase in *Scl6a3* mRNA levels (t(11) = 1,56, *p* = 0.15; Fig. 5b). As *Drd1a* mRNA expression was below the level of detection in the tegmentum of several animals, analysis of *Drd1a* was only performed in the PfCtx, Cpu and nAc. No difference was observed between WT and α-DB KO mice in *Drd1a* mRNA levels (genotype: F(1,32) = 1.1, *p* = 0.29; Fig. 5c). Similarly, *Drd2* expression did not differ between mouse groups in any brain areas (genotype: F(1, 44) = 1.3, *p* = 0.24; Fig. 5d). *Cnr1* mRNA levels were not significantly different between animal groups, although there was a trend towards increased expression in the PfCtx and decreased expression in the tegmentum of KO mice (genotype: F(1, 45) = 0.01, *p* = 0.9; tissue: F(3,45) = 0.06, post hoc *p* = 0.07; Fig. 5e). *Oprm1* expression was similar between genotypes in the PfCtx and tegmentum, but levels were significantly decreased in the Cpu and nAc of α-DB KO compared to WT animals (genotype: F(1,40) = 8.0, *p* = 0.007; all post hocs *p* < 0.05; Fig. 5f).

**Fig. 6:**
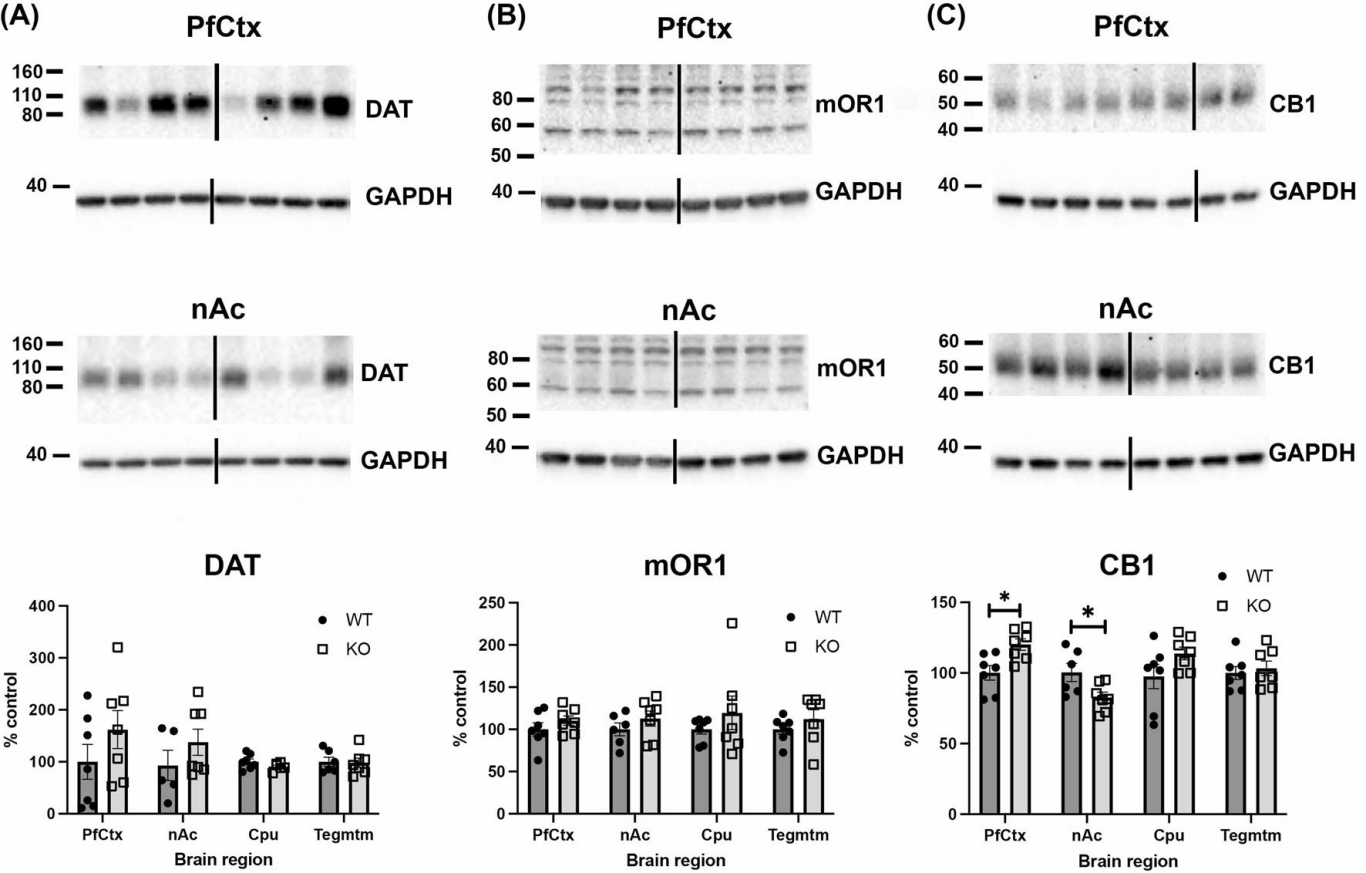
Western blots of dopamine transporter (DAT), mu opioid receptor 1 (mOR1) and cannabinoid receptor 1 (CB1) protein levels. DAT (**a**) and mOR1 (**b**) levels did not differ between WT and α-DB KO mice in the prefrontal cortex (PfCtx), nucleus accumbens (nAc), caudate-putamen (Cpu) or Tegmentum (Tegmtm). However, CB1 expression (**c**) was upregulated in the PfCtx and downregulated in the nAc of α-DB KO mice relative to WT mice. Numbers represent the molecular weight markers (kDa). Black lines represent non-continuous lanes loaded on the same gel. n = 7/group, *p < 0.05, two-way ANOVA with Sidak’s post-hoc

Based on the RT-qPCR results, brain tissues were processed by Western blotting to determine if protein levels of DAT, mOR1 and CB1 differed between animal groups. Levels of DAT were similar between WT and KO animals in all brain regions examined (genotype: F(1, 44) = 2.1, *p* = 0.16; Fig. 6a). The anti-mOR1 receptor detected two strong bands at 60 and 90 kDa. Quantification of the 60 kDa band, which most closely matched the predicted molecular weight of mOR1 [30], detected no differences between WT and KO mice (genotype: F(1,47) = 3.7, *p* = 0.06; Fig. 6b). However, CB1 protein levels were significantly higher in the PfCtx and lower in the nAc of KO mice compared to WT animals (genotype x area: F(3, 46) = 5.38, *p* = 0.003; all post hocs *p* < 0.05; Fig. 6c).

### No changes CB1 protein levels were observed in astrocytes

**Fig. 7:**
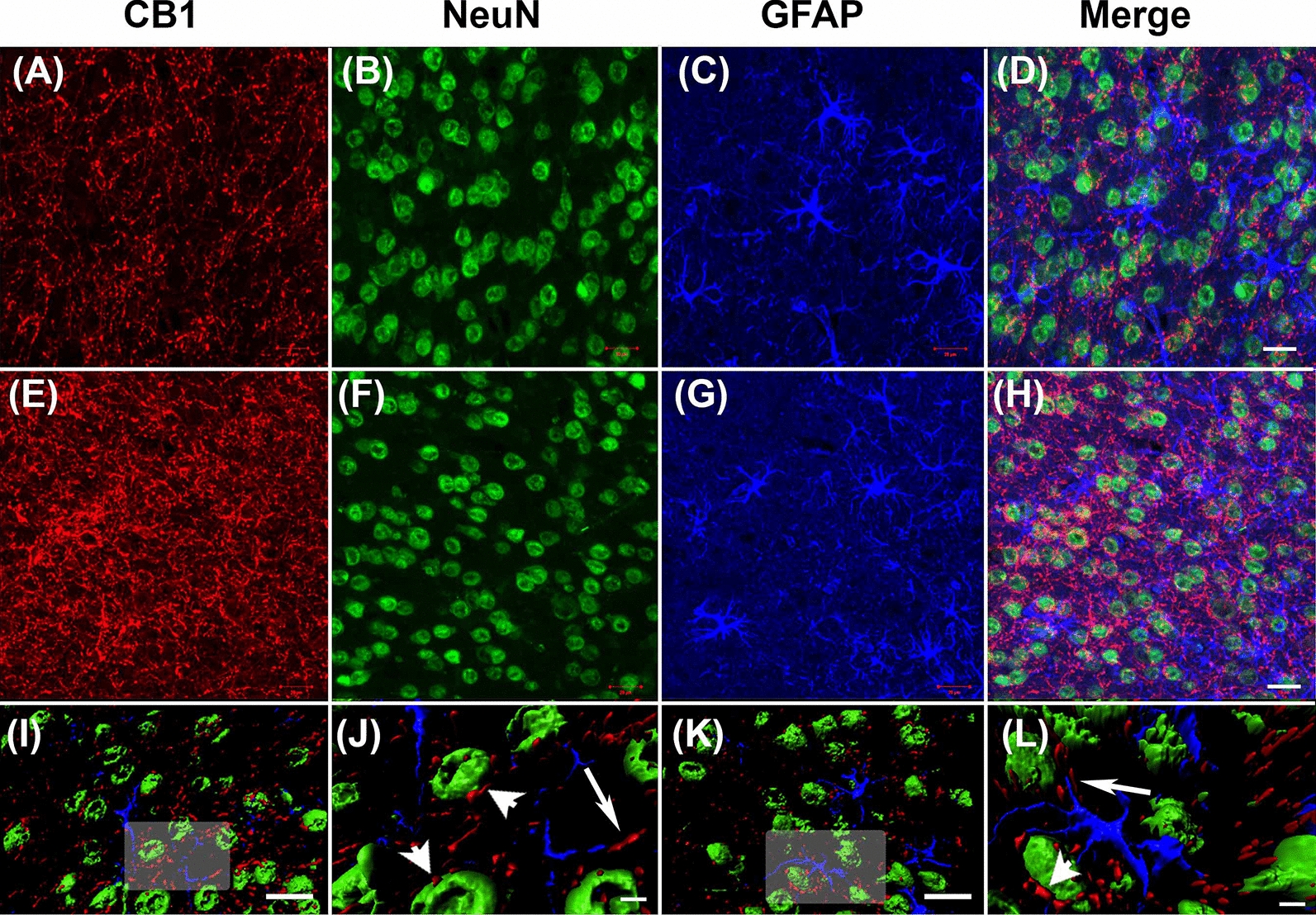
Photomicrographs of cannabinoid receptor 1 (CB1) expression in neurons and astrocytes. Confocal microscopy images of cortical brain sections triple labelled for CB1 (red), NeuN (green) and GFAP (blue) in WT (**a**–**d**, **i** and **j**) and α-DB KO mice (**d**–**h**, **k** and **l**). 3D reconstruction of confocal images (**i**–**l**) shows direct contact between CB1-positive staining and NeuN-positive cells (arrow heads), while little to no colocalization is noted between CB1 and GFAP (arrows). Images in **j** and **l** are magnifications of shaded areas in **i** and **k**. Scale bar **d**, **h**, **i** and **k** = 20 μm, **j** and **l** = 5 μm

Although CB1 is predominantly expressed in neurons, the receptor is also expressed in astrocytes [31, 32]. Because astrocyte dysfunction is reported in α-DB KO mice, brain tissues were processed for triple-labelling immunohistochemistry of CB1, NeuN and GFAP to determine if CB1 expression was specifically altered in the astrocytes of α-DB KO mice. CB1 expression appeared punctate within fibers surrounding NeuN-positive cells throughout the cortex in a distinct pattern to that of GFAP expression (Fig. 7a–h). 3D reconstruction of the confocal images found that NeuN-positive neurons made direct contact with CB1, while little contact was observed between GFAP-positive astrocytes and CB1 in either WT or α-DB KO mice (Fig. 7I-L). This suggests that the observed alterations in CB1 levels were not specific to astrocytes, but likely due to changes in neuronal expression.

## Discussion

Knockout of α-DB in the brain is associated with damaged astrocyte endfeet, altered extracellular matrix and increased blood–brain barrier permeability [11, 12]. This vascular dysfunction has potential consequences for neuronal function and associated behaviour. Here we report that α-DB KO mice had significantly increased motivation for an appetitive reward, while measures of executive function and extinction were unaffected. Increased motivation was associated with altered levels of CB1 in the PfCtx and nAc, which was not specific to GFAP-positive astrocytes.

Cognitive and behavioural impairments have been reported in both human and animal models of muscular dystrophies. A subset of DMD patients have lower IQ and exhibit deficits in language processing, learning and memory [33, 34]. The prevalence of attention-deficit hyperactivity disorders, autism spectrum disorders, and obsessive–compulsive disorders is also higher in DMD males [35]. The *mdx* mouse model of DMD shows faster acquisition in the PVD task and impaired acquisition and recall of fear-based memories, but no difference in spatial memory, cognitive flexibility or measures of motivation compared to WT animals [36, 37]. Interestingly, Lewon et al. found that *mdx* mice performed better than WT mice on operant learning and memory tasks that involved food reinforcement and speculated that this was due to differential effects of food deprivation on levels of motivation [38].

Our results suggest α-DB KO mice have elevated motivation for a hedonistic food reward, as evidenced by the high response rate in the PR16, ERC64 and FR64 tasks, which have a very low frequency of reward delivery. It is possible that the higher breakpoints may have been due to a perseverative behaviour, or that α-DB KO mice found the task associated non-consummatory stimuli (e.g. engagement with the touchscreen) to be rewarding. Indeed, ≥ 50% correct trial performance during reversal learning in the PVD task, which is a measure of relatively low perseveration and higher learning [27], was significantly lower in KO animals relative to WT mice. However, as WT and α-DB KO mice showed a similar pattern of learning in the PVD task and on the extinction task, the degree of the perseveration was not such that it precluded other aspects of learning. In addition, fully fed KO mice drank more milkshake than WT animals, indicating that they found the milkshake reward pleasurable. However, we also observed that latency to collect the reward did not differ between WT and KO animals in any of the tasks, which is unexpected in highly motivated animals [39]. α-DB KO mice have mild muscular dystrophy and display decreased grip strength and balance time compared to WT animals [6]. Thus, possible muscular defects may have masked a higher motivation-driven speed. However, in this case, KO mice would also be expected to make fewer total beam breaks and/or magazine entries, which was not observed. Thus, the results support a role of α-DB in appetitive motivation, although more work is needed to understand discrepancies between expected and observed motivation-related behaviours and the possible impact of α-DB KO on perseverative behaviours.

We also found that while both WT and KO mice ate more food as the ERC schedule became increasingly more demanding, only WT mice showed a corresponding decrease in total touches. In addition, α-DB KO animals consumed the same amount of milkshake as WT mice when food-restricted, but more milkshake when fully fed. This suggests that milkshake has both a high incentive and high hedonistic value for KO mice, but that these values are not sufficient to override homeostatic feeding. KO mice may also have a dysregulated appetite that drives them to consume more food in general, although *ad lib* food and water intake was similar between groups. Body weight did not differ between WT and KO animals at either the start or end of the experiment, but α-DB KO mice showed a trend towards greater weight gain over time than WT animals, which may reflect altered endogenous metabolism that has been reported in DMD and may contribute to the higher prevalence of obesity observed in adolescence [40, 41].

The consumption of palatable foods is related to both the hedonistic value or ‘liking’ ascribed to those items and to the incentive value or “wanting” of the food [20, 23]. Appetitive motivation is linked to DA signalling in the mesolimbic pathway and in particular, elevations in DA are observed in the nAc in response to intake of appetizing food and operant responding for food [20, 23]. Modulation of DA levels in the nAc affects food-reinforced responding but does not alter total or duration of food intake [42, 43]. By contrast, hedonistic feeding is related to the mesocortical DA pathway, and DA levels are increased in the PfCtx, but not in the nAc, before and during consumption of food in food-restricted animals. The hedonic and motivational impacts of rewarding foods are further modulated by activity of the eCB system [44]. In the mesocortical pathway, DA signalling is regulated via a negative feedback loop in which activation of GABA interneurons by DA inhibits the firing of glutamatergic neurons that project back to the VTA. At the same time, GABA binding to its receptors stimulates the release and retroactive diffusion of eCBs, where they bind to CB1 on presynaptic GABA neurons and block GABA release [24]. Ultimately, stimulation of CB1 increases DA levels in the PfCtx while antagonism of CB1 attenuates DA release induced by palatable foods [45, 46]. A similar mechanism also contributes to the eCB modulation of DA release in the nAc [47].

Based on the high level of motivation for the milkshake reward shown by α-DB KO mice, we hypothesized that DA signalling would be altered in the mesolimbic pathway of these animals. However, mRNA and protein levels of DA transporter and DA receptor 1 and 2 did not differ between WT and KO animals in any brain region examined. It may be that acute changes in gene expression were missed after re-feeding or between testing and tissue collection. Alternatively, elevated motivation may be related to a larger transient release of DA during operant responding that did not correspond to long-term changes in DA receptor expression. In support of this, CB1 expression was significantly lower in the nAc and higher in the PfCtx of KO animals. These findings are similar to previous studies that found a reduction in CB1 density in the nAc and upregulated CB1 binding in the cingulate cortex in animals with variable access to highly palatable food [48]. Therefore, the elevated appetitive motivation in the KO animals may be due in part to indirect modulation of DA signalling via alterations in CB1 expression. Although food restriction itself induces a downregulation in CB1, it is unlikely that this contributed to the observed changes in the α-DB KO mice because receptor levels return to normal during and up to an hour after food intake [24] and tissues were collected 6 weeks after *ad lib* feeding was re-introduced. However, additional experiments in food restricted vs fully fed WT and α-DB KO animals are needed to determine the potential effect of restriction on levels of CB1 expression and/or other proteins.

Finally, it is unclear why KO of α-DB leads to alterations of CB1 expression. Based on our current findings, we cannot discount the possible influence of behavioural training on the observed changes in CB1 and mOR1 expression between WT and α-DB KO mice. Additional comparisons with behaviourally naïve animals are necessary to establish if the differences in CB1 expression are an endogenous consequence of α-DB KO. However, both WT and KO animals underwent the same experimental manipulations, suggesting that differences in CB1 expression are likely related to genotype. Our results also suggest that there is minimal expression of CB1 in cortical astrocytes and therefore it is unlikely that changes in astrocyte structure or function contribute directly to the observed differences in CB1 levels. DAPC proteins help to stabilize GABA_A_ receptors in the postsynaptic neuron and receptor clusters are reduced in cerebellar and hippocampal neurons in *mdx* and double α- and β-DB KO animals [6, 49]. In addition, conditional KO of dystroglycan causes loss of cholecystokinin-expressing (CCK) GABAergic neurons in the cortex and hippocampus [50]. Interestingly, amongst the GABA interneuron populations, only CCK-positive cells express CB1. Therefore, KO of α-DB may disrupt the DAPC and destabilize GABAergic synapses, thereby altering associated levels of CB1. However, additional work is required to confirm the relationship between α-DB and the regulation of CB1 expression.

## Conclusion

In summary, our data suggest that α-DB and/or the DAPC contributes to the regulation of appetitive motivation and may provide additional insight into the neurobiology underlying DA signalling and related conditions, including behavioural disorders in muscular dystrophies.

## Supplementary Information

**Additional File 1: Figure S1.** Behavioural outputs of WT and α-DB KO mice on the PR tasks. The breakpoint of α-DB KO mice in the PR4 (a) and PR12 task (b) was significantly higher than WT animals. The majority of WT and KO mice demonstrated persistent responding for the duration of the PR4, PR8 and PR12 schedules (c). In the PR16 schedule, 33% and 42% of WT and KO mice, respectively, continued to engage with the task for the full 60 min. n = 12/group, *p < 0.05, two-tailed Student’s t-test.

**Additional File 1: Figure S2.** Behavioural outputs of WT and α-DB KO mice on the ERC and extinction tasks. Reward collection latencies (a), front and rear beam breaks/sec (b) and magazine entries/sec (c) did not differ between WT and KO mice in any of the ERC schedules. The number of trials completed during the extinction task (d) decreased significantly by day 4 in both WT and α-DB KO mice, however both groups showed a similar profile across time. N = 12/group. *p* < 0.0001 vs day 1, two-way repeated measures ANOVA.

## Data Availability

The datasets used and/or analysed during the current study are available from the corresponding author on reasonable request.

## Abbreviations

*Actb*: β-Actin
α-DB: α-Dystrobrevin
CB1 and * Cnr1*: Cannabinoid receptor 1
CCK: Cholecystokinin-expressing GABA neurons
Cpu: Caudate-putamen
DA: Dopamine
*Drd1a*: Dopamine receptor 1
*Drd2*: Dopamine receptor 2
DAT and *Scl6a3*: Dopamine transporter
DMD: Duchenne muscular dystrophy DMD
*Dtnbp1*: Dystrobrevin binding protein 1
DAPC: Dystrophin-associated protein complex
ERC: Effort-related choice
eCB: Endocannabinoid
FR: Fixed ratio task
GAPDH: Glyceraldehyde-3- phosphate dehydrogenase
KO: Knockout
mOR1 and *Oprm1*: Mu opioid receptor 1
nAc: Nucleus accumbens
NeuN: Neuronal nuclear protein
PfCtx: Prefrontal cortex
PR: Progressive ratio task
PVD: Pairwise visual discrimination
VTA: Ventral tegmental area
WT: Wildtype
