# Supplementary figures and images for: α-Dystrobrevin knockout mice have increased motivation for appetitive reward and altered brain cannabinoid receptor 1 expression
Source: Acta Neuropathol Commun. 2022 Aug 31;10:127. doi: 10.1186/s40478-022-01434-4 (PMC9434862; doi:10.1186/s40478-022-01434-4)

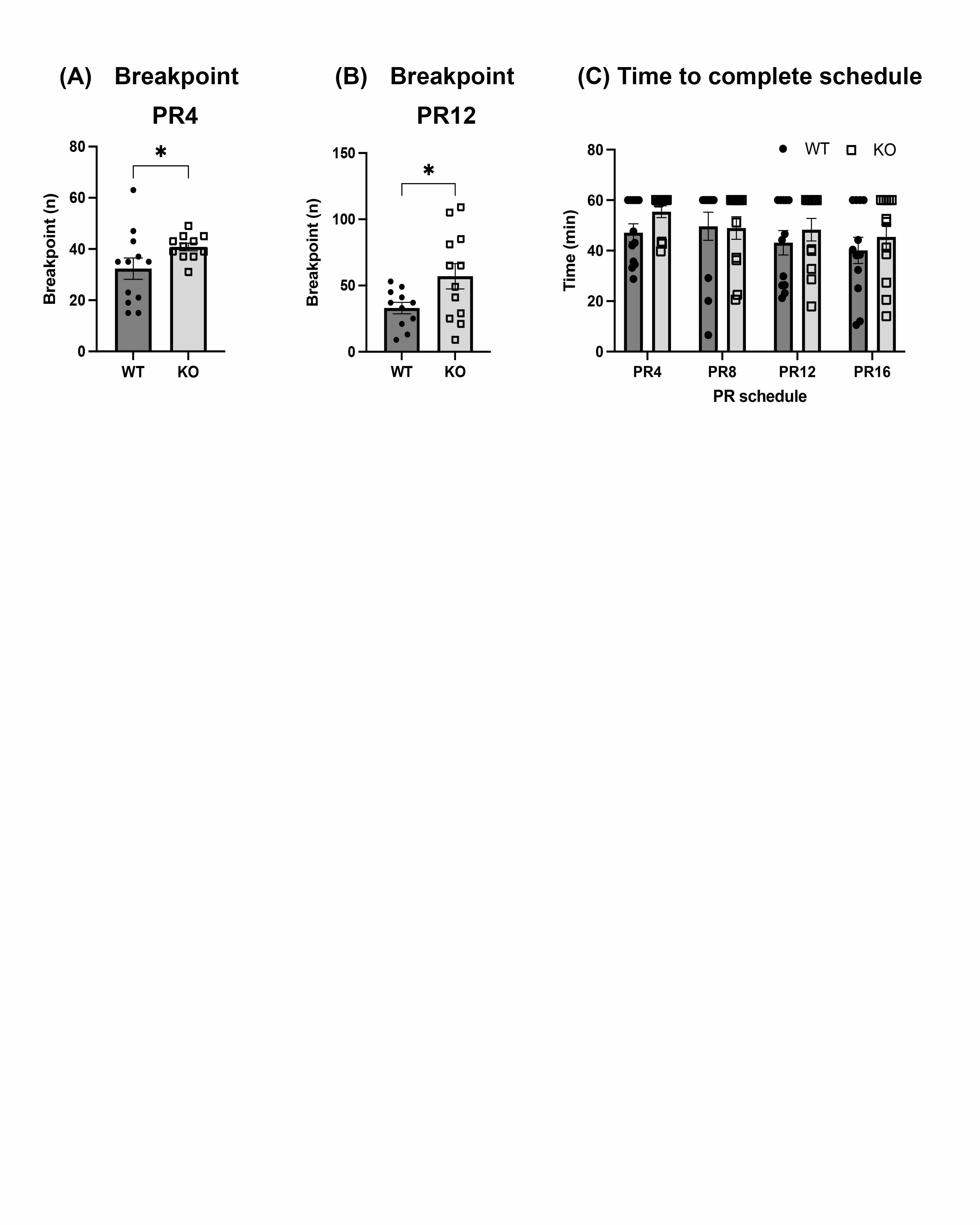

Supplement: Supplementary file 1 — Additional File 1: Figure S1. Behavioural outputs of WT and α-DB KO mice on the PR tasks. The breakpoint of α-DB KO mice in the PR4 (a) and PR12 task (b) was significantly higher than WT animals. The majority of WT and KO mice demonstrated persistent responding for the duration of the PR4, PR8 and PR12 schedules (c). In the PR16 schedule, 33% and 42% of WT and KO mice, respectively, continued to engage with the task for the full 60 min. n = 12/group, *p < 0.05, two-tailed Student’s t-test. [file 40478_2022_1434_MOESM1_ESM.jpg]

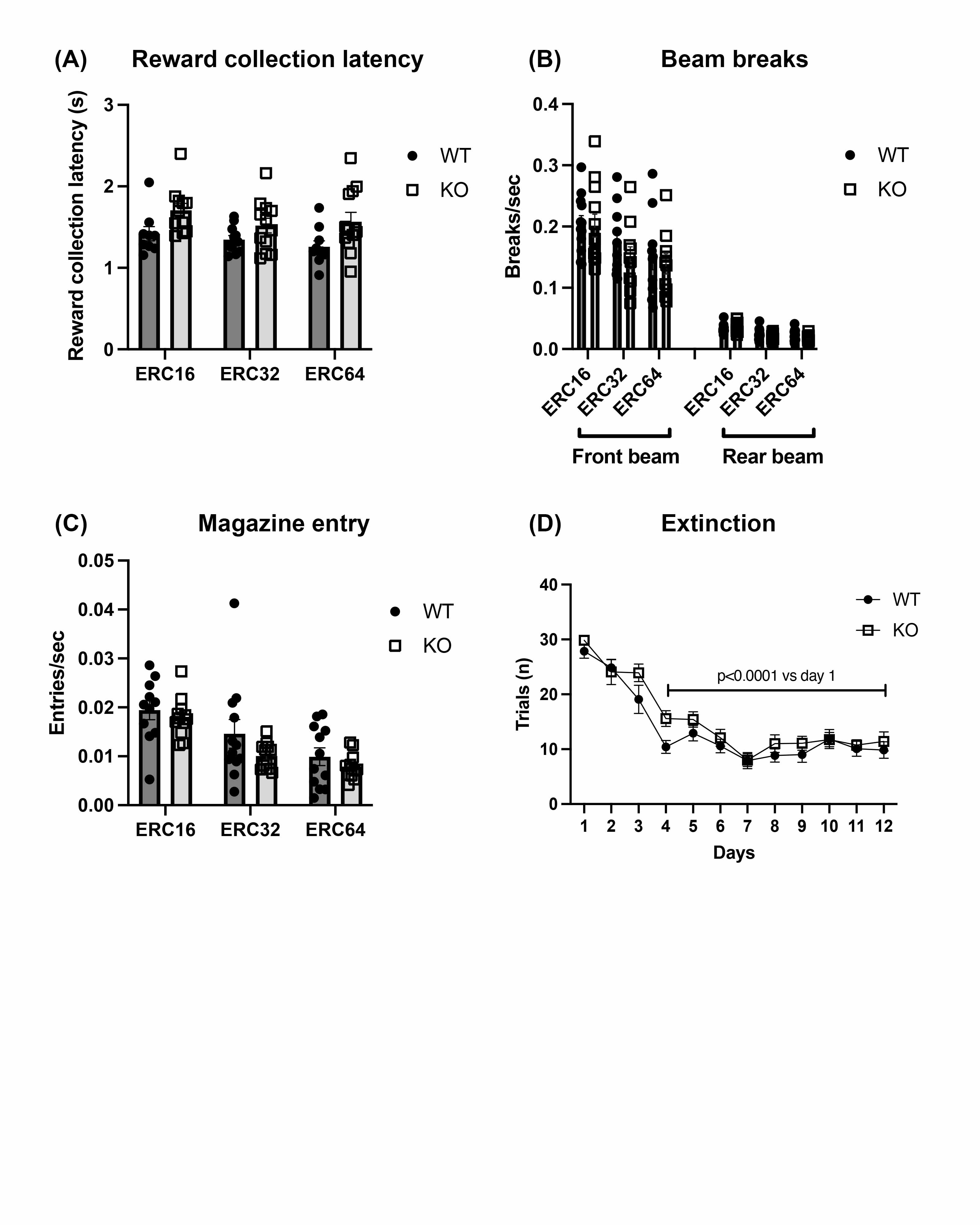

Supplement: Supplementary file 2 — Additional File 1: Figure S2. Behavioural outputs of WT and α-DB KO mice on the ERC and extinction tasks. Reward collection latencies (a), front and rear beam breaks/sec (b) and magazine entries/sec (c) did not differ between WT and KO mice in any of the ERC schedules. The number of trials completed during the extinction task (d) decreased significantly by day 4 in both WT and α-DB KO mice, however both groups showed a similar profile across time. N = 12/group. p < 0.0001 vs day 1, two-way repeated measures ANOVA. [file 40478_2022_1434_MOESM2_ESM.jpg]
